# Development and Validation of Ferroptosis- and Immune-Related lncRNAs Signatures for Breast Infiltrating Duct and Lobular Carcinoma

**DOI:** 10.3389/fonc.2022.844642

**Published:** 2022-04-04

**Authors:** Tao Wei, Ning Zhu, Weihua Jiang, Xiao-Liang Xing

**Affiliations:** ^1^ Department of Surgical Oncology, Urumqi Friendship Hospital, Urumqi, China; ^2^ School of Public Health and Laboratory Medicine, Hunan Provincial Key Laboratory for Synthetic Biology of Traditional Chinese Medicine, Hunan University of Medicine, Huaihua, China; ^3^ Department of Breast Surgery, The Affiliated Tumor Hospital of Xinjiang Medical University, Urumqi, China

**Keywords:** breast infiltrating duct and lobular carcinoma, ferroptosis, immune, lncRNAs, prognosis, diagnosis

## Abstract

**Background:**

Heterogeneity of breast cancer (BRCA) is significantly correlated with its prognosis. Target therapy for ferroptosis and immunity is a new cancer treatment option discovered in recent years. In the present study, we aimed to identify ferroptosis- and immune-related long non-coding RNAs (lncRNAs) to accurately predict the prognosis and diagnosis of patients with breast infiltrating duct and lobular carcinoma by integrated analyses.

**Methods:**

The corresponding data for the patients with breast infiltrating duct and lobular carcinoma by integrated analyses were obtained from The Cancer Genome Atlas (TCGA). Analyses of univariate and multivariate Cox regressions were used to identify the suitable candidate biomarkers.

**Results:**

We found that seven ferroptosis- and immune-related differentially expressed lncRNAs (FI-DELs) (AC007686.3, AC078883.1, ADAMTS9-AS1, AL035661.1, CBR3-AS1, FTX, and TMEM105) were correlated with the overall survival of patients with breast infiltrating duct and lobular carcinoma. The areas under the receiver operating characteristic (AUCs) value of the prognosis model were all over 0.6 in training, validation, and entire groups. The sensitivity and specificity of the diagnosis model was 87.84% and 97.06%, respectively.

**Conclusions:**

Through a series of bioinformatics analyses, we found that the seven FI-DELs could serve as prognostic and diagnostic biomarkers for patients with breast infiltrating duct and lobular carcinoma. However, whether these seven biomarkers could be really applied to the clinic requires further investigations.

## Introduction

Breast cancer (BRCA) is the most common type of malignant tumor. Globally, BRCA has very high incidence rates (1). It is established that there are almost 2.3 million new cases and 70 thousands deaths in 2020 (1). As a benefit from the development of BRCA diagnosis and treatment, the 5-year survival rate of BRCA patients has increased to 90% in recent years (2–4). However, BRCA is often diagnosed at later stage because it is asymptomatic early, which can lead to serious consequences (5). Previous studies have also demonstrated that there are significant differences in the prognosis of BRCA due to the heterogeneous of tumor cell (6, 7). Therefore, it is necessary and important to identify novel prognostic and diagnosis signatures for different types of BRCA to guide clinical practice and improve overall survival.

Previous studies indicated that BRCA easily metastasizes to other organs, such as the bones and lungs (5). For non-metastatic BRCA, the primary goal of treatment is to eradicate the tumor from the breast and regional lymph nodes and prevent metastatic recurrence (8). For metastatic BRCA, treatment is aimed at prolonging life and relieving symptoms. At present, metastatic BRCA remains incurable in almost all patients. Neoadjuvant/adjuvant therapy has been used primarily in metastatic breast cancer (8). Evidence indicated that the response to neoadjuvant treatment and the prognosis of BRCA are positively influenced by the tumor-infiltrating lymphocytes (9, 10). The development and progression of BRCA are closely related to the immune microenvironment, such as CD8^+^ and CD4^+^ T cells (11–13). Ferroptosis, an iron-dependent form of regulated cell death, is first proposed by Dixon in 2012 (14). Cumulative evidence indicted that the ferroptosis and immune system can regulate each other and participate in the development and progression of cancers (15–20).

Long non-coding RNA (lncRNA) is a class of non-coding RNA molecules with transcription length over 200 nt (21). Previous studies have found that lncRNAs are closely involved in the development and progression of several cancers (including BRCA) in various ways and may serve as specific biomarkers (22–25). In this study, we aimed to identify suitable ferroptosis- and immune-related lncRNAs as prognosis and diagnosis biomarkers for patients with breast infiltrating duct and lobular carcinoma by integrated analyses.

## Material and Methods

### Data Acquisition

The RNA-seq counts data and their corresponding clinical information for 1,215 samples (102 normal and 826 patients with breast infiltrating duct and lobular carcinoma) were downloaded from The Cancer Genome Atlas (TCGA) database (https://portal.gdc.cancer.gov/). The list of recognized ferroptosis- and immune-related genes were downloaded from the database of FerrDb and ImmPort, respectively. The annotated lncRNAs were downloaded from Gene Transfer Format (GTF) file (http://asia.ensembl.org).

### Candidate Biomarkers Identification

The DESeq2 package in R (3.6.1) was used to screen the differentially expressed genes with the following criterion adj. p < 0.05, |logFC| ≥ 0.5 and base mean ≥ 50. Co-expression analyses for the genes and lncRNAs were determined by Pearson analyses with the following criteria: p < 0.05 and R ≥ 0.3. After dividing the patients with breast infiltrating duct and lobular carcinoma into two groups depending on the median value, univariate and multivariate Cox regressions were used to identify the overall survival (OS)-related signatures.

### Prognosis and Diagnosis Model Construction

According to previous studies, we constructed the risk assessment model and the diagnosis model (26, 27). Risk value = (−1.08) × Exp (AC007686.3) + (−0.85) × Exp (AC078883.1) + (−1.03) × Exp (ADAMTS9-AS1) + (1.48) × Exp (AL035661.1) + (1.18) × Exp (CBR3-AS1) + (1.56) × Exp (FTX) + (0.86) × Exp (TMEM105). The Logit value = 1.023 + (−0.150) × Exp (AC007686.3) + (−0.072) × Exp (AC078883.1) + (−0.132) × Exp (ADAMTS9-AS1) + (0.051) × Exp (AL035661.1) + (0.087) × Exp (CBR3-AS1) + (−0.104) × Exp (FTX) + (0.053) × Exp (TMEM105). The Express (Exp) values of the candidate biomarkers were obtained from the normalized value of DESeq2 analyses.

### Principal Component Analyses and Enrichment Analyses

Principal component analyses (PCAs) were conducted to reduce dimension and visualize the patients with breast infiltrating duct and lobular carcinoma with different risk values in R (3.6.1).

DAVID bioinformatics Resource 6.8 was used to perform the Gene Ontology and Kyoto Encyclopedia of Genes and Genomes analyses with the default parameters (28).

### Statistical Analyses

A repeated measures ANOVA followed by unpaired two-tailed Student’s t-test was used as indicated. All results are expressed as mean ± SEM.

## Results

### Identification of Ferroptosis- and Immune-Related lncRNAs as Biomarkers

**Figure 1:**
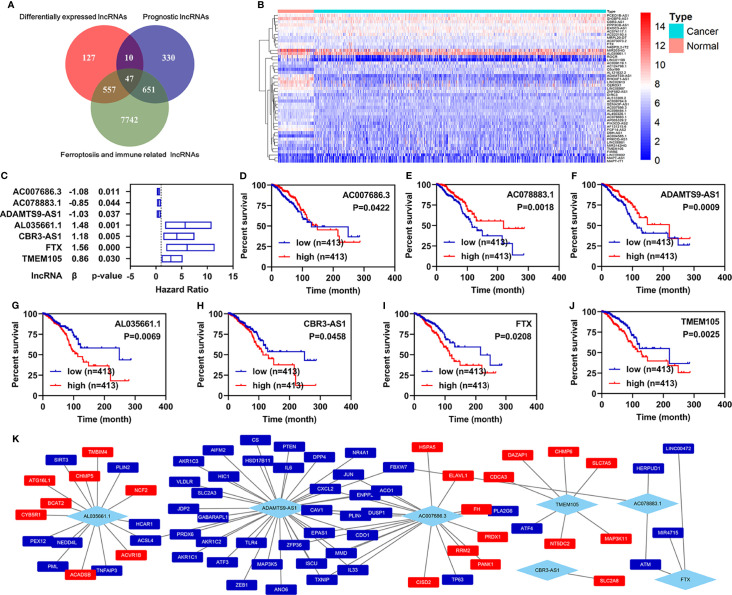
Identification of ferroptosis- and immune-related lncRNAs as biomarkers. **(A)** Venn diagram to identify the common lncRNAs of differentially expressed lncRNAs, ferroptosis- and immune-related lncRNAs, and prognostic lncRNAs. **(B)** Heatmap of 47 common lncRNAs identified by Venn analyses. **(C)** Multivariate Cox regression analyses illustrated that seven FI-DELs were correlated with the OS of patients with breast infiltrating duct and lobular carcinoma independently. **(D–J)** Overall survival curve of these seven FI-DELs. **(K)** The correlation of these seven FI-DELs with the FI-DGEs.

First, 16,901 lncRNAs were identified by analyzing the RNA-seq data from patients with breast infiltrating duct and lobular carcinoma, of which 741 lncRNAs were differentially expressed between the normal and the cancer patients. A total of 1,038 prognostic lncRNAs were identified by univariate Cox regression analyses in all patients with breast infiltrating duct and lobular carcinoma. To obtain ferroptosis- and immune-related lncRNAs, we downloaded 329 ferroptosis-related genes and 2,483 immune-related genes from the database of FerrDb and ImmPort, respectively. We then performed co-expression analyses for these ferroptosis- and immune-related genes with the lncRNAs and found that 8,997 ferroptosis- and immune-related lncRNAs correlated with 322 ferroptosis- and immune-related genes. Finally, we found that 47 ferroptosis- and immune-related differentially expressed lncRNAs (FI-DELs) were present among differentially expressed lncRNAs, prognostic lncRNAs, and ferroptosis- and immune-related lncRNAs (**Figure 1A**). The expression of these 47 FI-DELs are shown in **Figure 1B**. To construct a risk assessment model and further verify it, 826 patients with breast infiltrating duct and lobular carcinoma were randomly separated into training (n=413) or validation (n=413) group. **Table 1** presents the detail clinical characteristics of these patients.

**Table 1:** Clinical characteristic of patients in the training and validation groups.

Variables	Training group (n=413)		Validation group (n=413)	
	No.	%	No.	%
**Age**				
≤60	237	57.38%	240	58.11%
>60	176	42.62%	173	41.89%
**Stage**				
I	86	20.82%	62	15.01%
II	225	54.48%	250	60.53%
III	83	20.10%	83	20.10%
IV	12	2.91%	6	1.45%
X	7	1.69%	12	2.91%
**T**				
T1	129	31.23%	104	25.18%
T2	229	55.45%	263	63.68%
T3	36	8.72%	31	7.51%
T4	18	4.36%	14	3.39%
TX	1	0.24%	1	0.24%
**N**				
N0	191	46.25%	188	45.52%
N1	143	34.62%	146	35.35%
N2	47	11.38%	54	13.08%
N3	24	5.81%	17	4.12%
NX	8	1.94%	8	1.94%
**M**				
M0	356	86.20%	357	86.44%
M1	12	2.91%	8	1.94%
MX	45	10.90%	48	11.62%
**Gender**				
Female	406	98.30%	409	99.03%
Male	7	1.70%	4	0.97%

Through multivariate Cox regression analyses in the training group, we found that seven FI-DELs were correlated independently with the overall survival of patients with breast infiltrating duct and lobular carcinoma (**Figures 1C–J**). The interactions of these seven FI-DELs with the FI-DEGs are displayed in **Figure 1K**.

### Construction and Validation of the Prognostic Model

**Figure 2:**
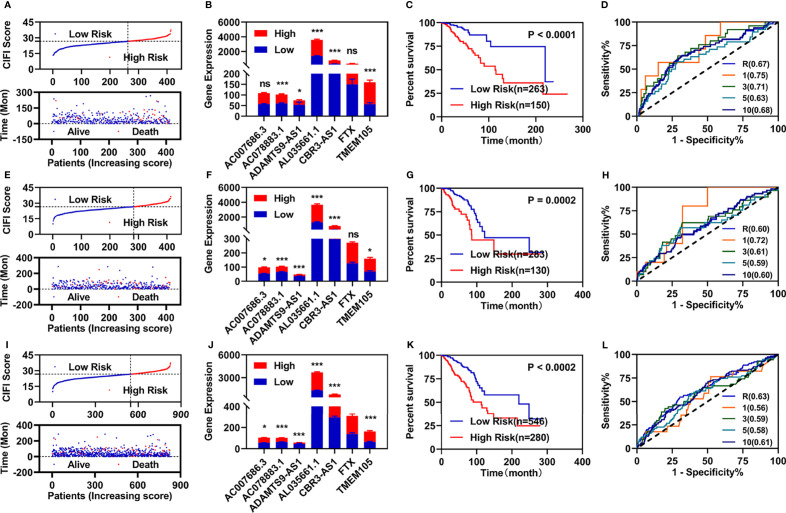
Developments and validations of prognosis model. **(A, E, I)** Risk value (up) and survival status (down) for each patients in the training group **(A)**, validation group **(E)**, and entire group **(I)**. **(B, F, J)** Expression of these seven candidate biomarkers in the training group **(B)**, validation group **(F)**, and entire group **(J)**. **(C, G, K)** Overall survival curve of patients with breast infiltrating duct and lobular carcinoma with different risk value in the training group **(C)**, validation group **(G)**, and entire group **(J)**. **(D, H, L)** ROC curve of risk assessment model in the training group **(D)**, validation group **(H)**, and entire group **(L)**. *p < 0.05, ***p < 0.001. ns, no significance.

According to previous studies, we first constructed a risk assessment model using these seven FI-DELs in the training group. The patients were further divided into high- and low-risk groups by the Youden index as the optimal cutoff value (**Supplementary Figure S1**). The risk value (up) and survival status (down) for each patients in the training group was displayed in **Figure 2A**. The expression of AC078883.1 and ADAMTS9-AS1 were significantly decreased, while the expression of the AL035661.1, CBR3-AS1, FTX, and TMEM105 were significantly increased in the patient with breast infiltrating duct and lobular carcinoma with high-risk value (**Figure 2B**). Patients with breast infiltrating duct and lobular carcinoma with high-risk value had a higher probability of death than these patients with the low-risk value (**Figure 2C**). As shown in **Figure 2D**, the area under ROC curve (AUC) reached 0.67. The time-dependent AUC reached 0.75 at 1 year, 0.71 at 3 years, 0.63 at 5 years, and 0.68 at 10 years.

We then performed similar investigations for the patients in the validation and entire groups. Similar results are shown in **Figures 2E–L**. All AUC values of the prognosis model in the training, validation, and entire group were over 0.60.

### Independent Prognostic Value of Risk Assessment Model

**Figure 3:**
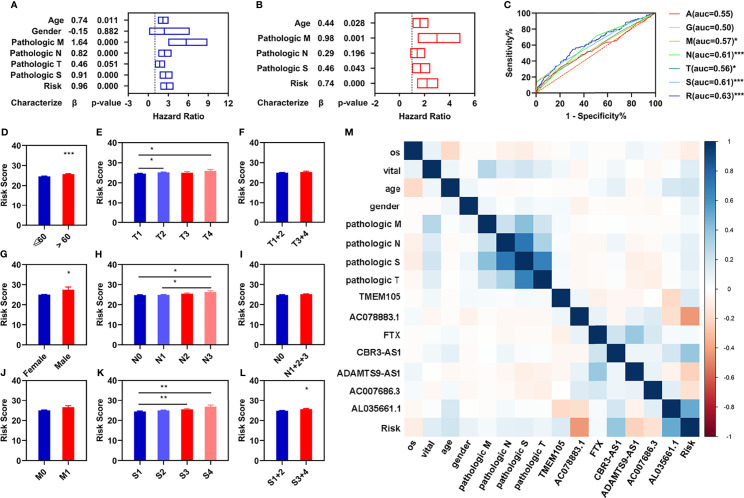
Independent prognostic values of these four FI-DELs. **(A)** Univariate Cox regression analyses illustrated four clinical characteristics and risk assessment model were correlated with OS of patients. **(B)** Multivariate Cox regression analyses illustrated that three clinical characteristics and risk assessment model were correlated with the OS of patients independently. **(C)** ROC curve of different clinical characteristics and risk assessment model. **(D–L)** Comparison of the risk score in different clinical characteristics groups. **(M)** Correlation analyses of these seven FI-DELs with the clinical characteristics. *p < 0.05, **p < 0.01, ***p < 0.001.

To determine whether the risk assessment model was an independent prognostic factor for the patients with breast infiltrating duct and lobular carcinoma, univariate and multivariate Cox regression analyses were performed among the clinical characteristics and the prognostic model. The results of the univariate Cox regression showed that the risk assessment model was significantly associated with the OS in the training, validation, and entire groups (**Figure 3A**; **Supplementary Figures S2A, D**). The risk assessment model remained an independent predictor of OS in multivariate Cox regression analyses (**Figure 3B**; **Supplementary Figures S2B, E**). Additionally, the ROC curve analyses showed that the AUC value of the risk assessment model in the training group, validation group, and entire group were 0.67, 0.60, and 0.63 respectively (**Figures 2D, H, L**), which were higher than the AUC values of other prognostic factors (**Figure 3C**; **Supplementary Figures S2C, F**).

To investigate the relationships of these seven FI-DELs with different clinical characteristics, we performed differential expression analyses and found that the risk values displayed significant difference among the patients with different age, gender, pathological TN, and pathological stage (**Figures 3D–L**). We further performed the correlation analyses and found that AC078883.1, CBR3-AS1, and AL035661.1 were significantly correlated with the risk value (**Figure 3M**).

### The Immune Cell Infiltration Landscape in Breast Cancer

**Figure 4:**
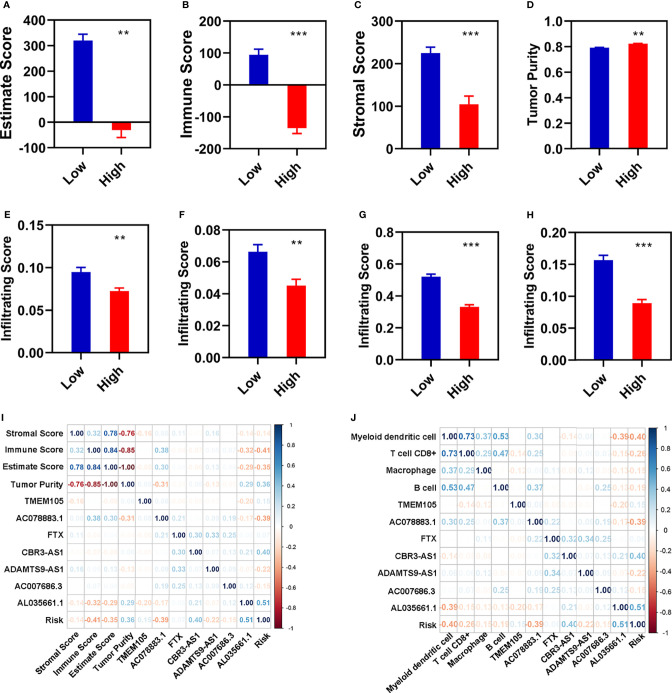
Correlation analyses of risk assessment model with the immunity. **(A–D)** Comparison of the estimate score **(A)**, immune score **(B)**, stromal score **(C)**, and tumor purity **(D)** between the patients with different risk value. **(E–H)** Comparison of the infiltrating score between the patients with different risk value. **(E)** B cell. **(F)** Macrophage. **(G)** Myeloid dendritic cell. **(H)** T cell CD8^+^. **(I, J)** Correlation analyses of these seven FI-DELs with the ESTIMATE score **(I)** and infiltrating score **(J)**. **p < 0.01, ***p < 0.001.

To further explore the relationship between FI-DELs and the immunity, we evaluated the immunity status using ESTIMATE in R (3.6.1). The estimate and stromal scores were significantly decreased, while the immune score and tumor purity was significantly increased in patients with breast infiltrating duct and lobular carcinoma (**Supplementary Figures S3A–D**). The estimate, immune, and stromal scores were significantly decreased, while the tumor purity was significantly increased in patients with breast infiltrating duct and lobular carcinoma with different risk score (**Figures 4A–D**). The correlation of immune status with these seven FI-DELs is displayed in **Figure 4I**.

Subsequently, we used the TIMER algorithm to investigate the landscape of the immune cell infiltration. The infiltration score of the macrophage, myeloid dendritic cell, and T cell CD8^+^ were significantly decreased, while the infiltration score of B cell was significantly increased in the patients with breast infiltrating duct and lobular carcinoma (**Supplementary Figures S3E–H**). The infiltration score of these four immune cells and factors were significantly decreased in patients with breast infiltrating duct and lobular carcinoma with high-risk value (**Figures 4E–H**). The correlation of immune cells and factors with these seven FI-DELs is displayed in **Figure 4J**.

### Immune-Related Pathways Were Activated in the Low-Risk Group

**Figure 5:**
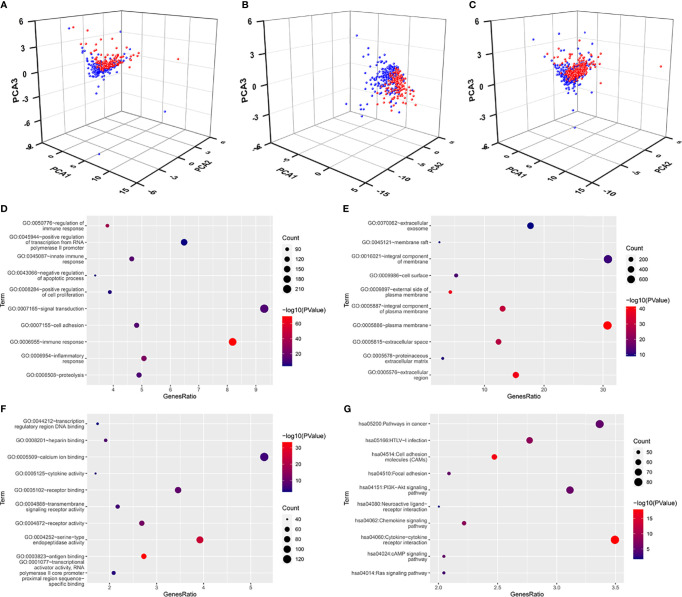
Principal component analyses and enrichment analyses. **(A–C)** Principal component analyses (PCA) plots depicted distinct distribution of high- and low-risk groups based on these seven FI-DEGs in the training group **(A)**, validation group **(B)**, and entire group **(C)**. Blue means low-risk group. Red means high-risk group. **(D–F)** Top 10 significantly enriched GO. **(D)** For BP. **(E)** For CC. **(F)** For MF. **(G)** Top 10 significantly enriched KEGG.

To explore the distribution of the patients with different risk score, we performed the PCA analyses using these seven FI-DELs and found that the patients with breast infiltrating duct and lobular carcinoma with low-risk value could be well separated from these patients with high-risk value in the training, validation, and entire groups (**Figures 5A–C**).

We subsequently performed differentially expressed analyses for these patients with different risk score. A total of 2,582 genes (660 upregulated DEGs and 1,922 downregulated DGEs) were differentially expressed between the low- and high-risk group (**Supplementary Figure SS4**). An enrichment analyses was carried out for these 2,582 DEGs using DAVID. A total of 563 BP, 89 CC, and 143 MF were enriched as measured by the false discovery rate (FDR) value <0.05 (**Supplementary Table S1**), and the top 10 of these GOs are displayed in **Figures 5D–F**, including several immune response GO term. KEGG analyses showed that 110 pathways were enriched as measured by the FDR value <0.05 (**Supplementary Table S2**), the top 10 of these pathways are displayed in **Figure 5G**, including ferroptosis- and immune-related pathway PI3K-Akt signaling pathway.

### Construction of a Diagnostic Model

**Figure 6:**
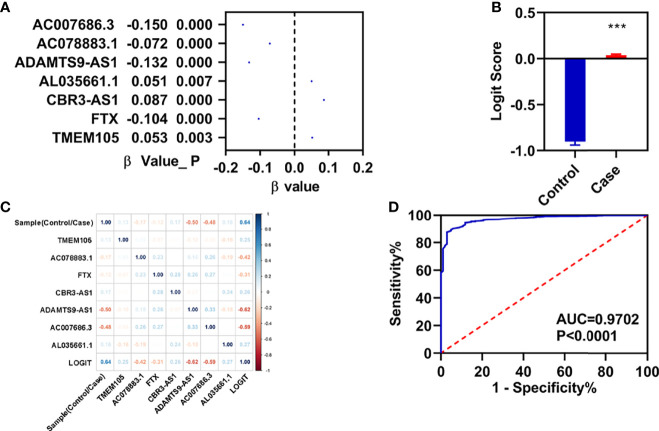
Construction of diagnosis model. **(A)** β-value of these seven FI-DELs analyzed by stepwise logistic regression. **(B)** Diagnosis values between normal and patients with breast infiltrating duct and lobular carcinoma. **(C)** Correlation analyses of these seven FI-DELs with diagnosis values. **(D)** ROC curves of the diagnostic model. ***p < 0.001.

To know the role in the diagnosis of patients with breast infiltrating duct and lobular carcinoma, we performed a stepwise logistic regression analyses for these seven FI-DELs (**Figure 6A**). The logit score of patients was significantly higher than that of the normal (**Figure 6B**). Correlation analyses showed that the logit score was significantly associated with AC078883.1, FTX, ADAMTS9-AS1, and AC007686.3 (**Figure 6C**). The sensitivity and specificity of the diagnose model were 87.53% and 97.06%, respectively (**Table 2**). We also plot the ROC curve of the diagnose model, and the AUC value was 0.9702 (**Figure 6C**).

**Table 2:** Sensitivity and specificity of diagnosis model.

	Real Cancer	Real Normal
Predicted Cancer	723	3
Predicted Normal	103	99
Total	826	102
Correct	723	99
Sensitivity	0.8753	
Specificity		0.9706

## Discussions

Because BRCA is often asymptomatic in early stages, most patients were diagnosed in later stages (5). In addition, previous studies have shown that the heterogeneity of BRCA is significantly correlated with its prognosis. Therefore, it is important to screen new prognostic and diagnostic biomarkers for different types of breast cancer to guide clinical practice (6, 7).

In the present study, we aimed to identify suitable ferroptosis- and immune-related lncRNAs as prognosis and diagnosis biomarkers for patients with breast infiltrating duct and lobular carcinoma by integrated analyses. Finally, we found that AC007686.3, AC078883.1, ADAMTS9-AS1, AL035661.1, CBR3-AS1, FTX, and TMEM105 were significantly associated with the OS of patients with breast infiltrating duct and lobular carcinoma. The risk assessment model and diagnosis model could predict the outcome of these patients. In addition, we found that the expression ADAMTS9-AS1 was decreased significantly, which demonstrated in previous studies as suppressor in BRCA cell invasion, proliferation, and the aggression (29, 30). In another research, Chen et al. also found that ADAMTS9-AS1, as prognostic biomarker, could promote cell proliferation and EMT in colorectal cancer (31). These results suggested that ADAMTS9-AS1 may play different roles for different types of cancers (29–31).

Fang et al. found that AL035661.1 was upregulated in renal cell carcinoma and positively associated with the poor prognosis (32). Similarly, we also found that the expression of AL035661.1 was decreased in patients with breast infiltrating duct and lobular carcinoma, and these patients with high expression of AL035661.1 exhibited worse OS. However, Lv et al. found that the expression of AL035661.1 was downregulated and could serve as prognosis biomarkers of hepatocellular carcinoma (33). These results indicated that AL035661.1 was closely related to the development and progression of several cancers (32, 33) and also increased the possibility of AL035661.1 as prognosis biomarker for BRCA patients with breast infiltrating duct and lobular carcinoma.

Previous studies have demonstrated that CBR3-AS1 could promote lung adenocarcinoma cell proliferation, migration, and invasion (34, 35). In BRCA, several studies have also demonstrated that the expression of CBR3-AS was upregulated in BRCA tissues and cells (36, 37). Those patients with high expression displayed poor OS (36, 37). Consistent with previous studies, we also found that CBR3-AS was increased and negatively significantly associated with the OS. Our present study reinforced the potential role of CBR3-AS1 as a therapeutic target and potential prognostic for breast cancer, especially for these BRCA patients with infiltrating duct and lobular carcinoma.

LncRNA FTX was first identified in Xist gene locus. Evidence indicated that FTX could promote the migration, proliferation, and invasion for several caners, including colorectal cancer, gastric cancer, and lung cancer (38–40). In this study, we found that the expression of FTX was downregulated. However, the patients with low expression of FTX exhibited better OS. Therefore, we hypothesized that the FTX expression was just correlated with the OS of BRCA patients. The aberrant expression of FTX could be a result of the progression of cancer.

For AC007686.3, AC078883.1, and TMEM105, although there were no reported relationships between them and cancer at present, our research suggested that they were closely related to BRCA cancer. Whether their abnormal expressions were the cause or the compensatory results remains to be further studied. In the future, we will also carry out functional studies in the further study.

## Conclusions

Target therapy for ferroptosis and immunity are new cancer treatment options discovered in recent years. In the present study, through a series of bioinformatics analyses, we found that seven FI-DELs could serve as prognostic and diagnostic biomarkers for patients with breast infiltrating duct and lobular carcinoma. However, whether these seven biomarkers could be really applied to the clinic requires further investigations.

## Data Availability Statement

Publicly available datasets were analyzed in this study. These data can be found here: https://portal.gdc.cancer.gov/.

## Author Contributions

TW and NZ conceived and designed the experiments. XX performed the analyses. WJ helped in the analysis of the data. XX wrote the paper. All authors contributed to the article and approved the submitted version.

## Funding

This project was financially supported by Foundation of Hunan University of Medicine (2020122004 and 20KJPY02).

## Conflict of Interest

The authors declare that the research was conducted in the absence of any commercial or financial relationships that could be construed as a potential conflict of interest.

## Publisher’s Note

All claims expressed in this article are solely those of the authors and do not necessarily represent those of their affiliated organizations, or those of the publisher, the editors and the reviewers. Any product that may be evaluated in this article, or claim that may be made by its manufacturer, is not guaranteed or endorsed by the publisher.
