# Supplementary material for: Development and Validation of Ferroptosis- and Immune-Related lncRNAs Signatures for Breast Infiltrating Duct and Lobular Carcinoma
Source: Front Oncol. 2022 Apr 4;12:844642. doi: 10.3389/fonc.2022.844642 (PMC9015165; doi:10.3389/fonc.2022.844642)
Supplement: Supplementary file 1 [file DataSheet_1.pdf]

Development and Validation of Ferroptosis- and Immune-related lncRNAs Signatures for Breast Infiltrating Duct and Lobular Carcinoma

Tao Wei[1#], Ning Zhu[2#], Weihua Jiang[3], Xiao-Liang Xing[2]

[1]Urumqi Friendship Hospital, Urumqi 830000, Xinjiang, China.

[2]School of Public Health and Laboratory Medicine, Hunan University of Medicine, Huaihua 418000, Hunan, P. R. China.

[3]The Affiliated Tumor Hospital of Xinjiang Medical University, Urumqi 830000, Xinjiang, China.

[#]Contributed equally to this work.

*Correspondence: Xiao-Liang Xing, xiaoliangxinghnm@126.com.

Supplementary: 4 figures and 2 tables

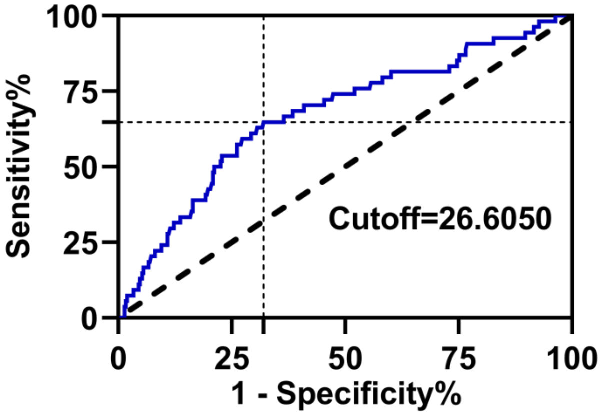

Supplementary Figure 1 The Youden index as the optimal cut-off value

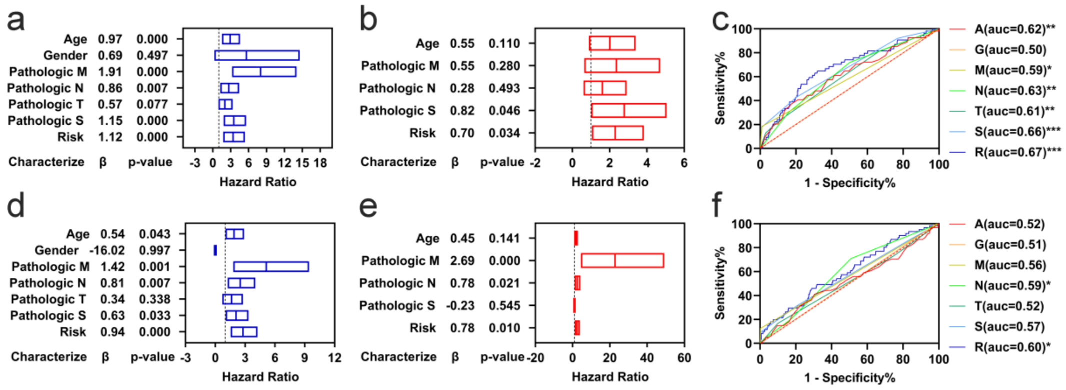

Supplementary Figure 2 Independent prognostic values of those 4 FI-DELs in training group and validation group.

a, d, Univariate Cox regression analyses illustrated four clinical characteristics and risk assessment model were correlated with OS of patients. a, for training group. d, for validation group. **b, e,** Multivariate Cox regression analyses illustrated three clinical characteristics and risk assessment model were correlated with the OS of patients independently. b, for training group. c, for validation group. **c, f** ROC curve of different clinical characteristics and risk assessment model. c, for training group. f, for validation group.

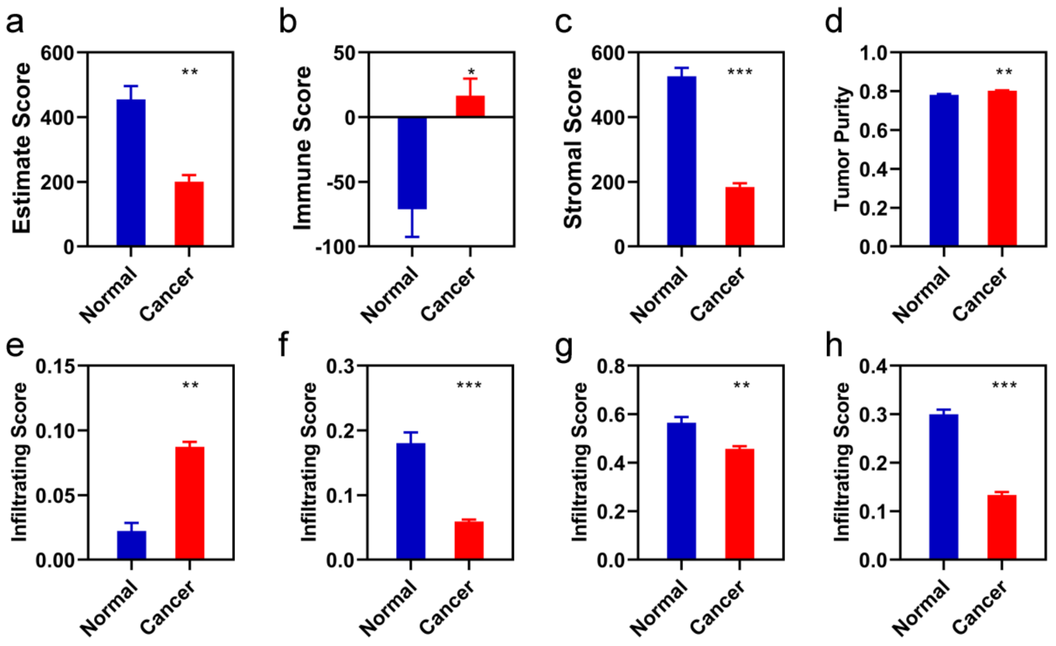

Supplementary Figure 3 Correlation analyses of risk assessment model with the immunity.

a-d, Comparison of the estimate score (a), immune score (b), stromal score (c), and tumor purity (d) between normal and cancer. **e-h,** Comparison of the infiltrating score between normal and cancer. e, B cell. f, Macrophage. g, Myeloid dendritic cell. h, T cell $CD8^+$. * $p < 0.05$, ** $p < 0.01$, *** $p < 0.001$.

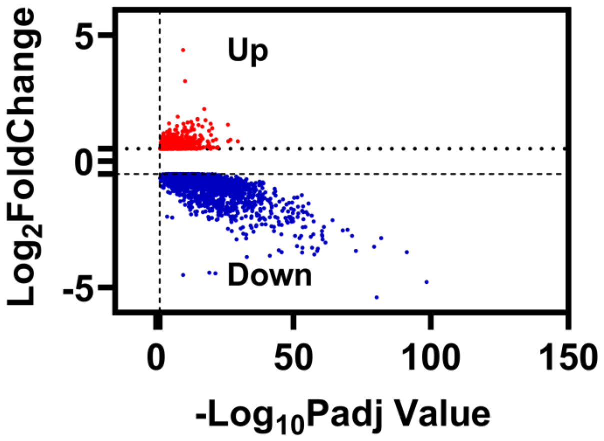

Supplementary Figure 4 Volcano plot of DEGs between the patients with different risk score.

Supplementary table 1 Significantly enriched GO term

Term		Count	FDR
BP	immune response	192	0.0000
	regulation of immune response	89	0.0000
	complement activation, classical pathway	65	0.0000
	complement activation	58	0.0000
	inflammatory response	119	0.0000
	receptor-mediated endocytosis	76	0.0000
	Fc-gamma receptor signaling pathway involved in phagocytosis	58	0.0000
	adaptive immune response	59	0.0000
	B cell receptor signaling pathway	34	0.0000
	innate immune response	109	0.0000
	T cell costimulation	38	0.0000
	positive regulation of T cell proliferation	33	0.0000
	cell adhesion	113	0.0000
	cell surface receptor signaling pathway	78	0.0000
	signal transduction	218	0.0000
	Fc-epsilon receptor signaling pathway	58	0.0000
	transmembrane receptor protein tyrosine kinase signaling pathway	40	0.0000
	proteolysis	115	0.0000
	response to lipopolysaccharide	54	0.0000
	phagocytosis, recognition	19	0.0000
	chemotaxis	42	0.0000

	positive regulation of ERK1 and ERK2 cascade	52	0.0000
	phagocytosis, engulfment	20	0.0000
	chemokine-mediated signaling pathway	29	0.0000
	positive regulation of B cell activation	17	0.0000
	cell-cell signaling	64	0.0000
	positive regulation of gene expression	63	0.0000
	cellular response to interleukin-1	27	0.0000
	cellular response to tumor necrosis factor	35	0.0000
	T cell activation	21	0.0000
	T cell receptor signaling pathway	41	0.0000
	leukocyte migration	36	0.0000
	positive regulation of inflammatory response	26	0.0000
	antigen processing and presentation of peptide or polysaccharide antigen via MHC class II	12	0.0000
	positive regulation of protein kinase B signaling	28	0.0001
	cell chemotaxis	24	0.0001
	positive regulation of cell proliferation	91	0.0001
	cellular response to interferon-gamma	22	0.0001
	extracellular matrix organization	48	0.0001
	cellular response to retinoic acid	24	0.0002
	cellular response to lipopolysaccharide	32	0.0003
	regulation of cell proliferation	44	0.0006
	neutrophil chemotaxis	22	0.0011
	negative regulation of immune response	9	0.0012
	central nervous system development	32	0.0012
	T cell differentiation	14	0.0012
	peptidyl-tyrosine autophosphorylation	16	0.0021
	peptidyl-tyrosine phosphorylation	37	0.0022
	positive regulation of interleukin-4 production	11	0.0026
	negative regulation of interferon-gamma production	13	0.0028
	angiogenesis	48	0.0028
	interferon-gamma-mediated signaling pathway	22	0.0032
	negative regulation of T cell proliferation	15	0.0032
	estrous cycle	10	0.0034
	dendritic cell chemotaxis	10	0.0034
	positive regulation of cytosolic calcium ion concentration	33	0.0039
	phosphatidylinositol phosphorylation	26	0.0039
	cellular defense response	20	0.0039
	humoral immune response	19	0.0039
	positive regulation of cell migration	41	0.0044
	T cell chemotaxis	7	0.0045
	MAPK cascade	53	0.0049
	skeletal system development	33	0.0055

	positive regulation of phosphatidylinositol 3-kinase signaling	20	0.0072
	positive regulation of smooth muscle cell proliferation	19	0.0073
	positive regulation of epithelial cell proliferation	19	0.0073
	positive regulation of transcription from RNA polymerase II promoter	152	0.0075
	regulation of cell shape	33	0.0078
	hair follicle morphogenesis	12	0.0078
	myeloid dendritic cell differentiation	10	0.0082
	positive regulation of interferon-gamma production	16	0.0090
	positive regulation of interleukin-2 biosynthetic process	8	0.0090
	brown fat cell differentiation	13	0.0093
	phosphatidylinositol-mediated signaling	27	0.0094
	regulation of phosphatidylinositol 3-kinase signaling	22	0.0099
	monocyte chemotaxis	15	0.0109
	negative regulation of Wnt signaling pathway	17	0.0109
	positive regulation of monocyte chemotaxis	9	0.0112
	negative regulation of apoptotic process	79	0.0129
	defense response to bacterium	33	0.0134
	platelet degranulation	26	0.0136
	skeletal muscle cell differentiation	16	0.0174
	negative regulation of cell proliferation	70	0.0174
	apoptotic signaling pathway	20	0.0196
	immunological synapse formation	7	0.0198
	negative regulation of B cell activation	6	0.0207
	hyaluronan biosynthetic process	6	0.0207
	heterophilic cell-cell adhesion via plasma membrane cell adhesion molecules	16	0.0207
	cell recognition	10	0.0251
	glial cell differentiation	8	0.0254
	response to cold	13	0.0266
	platelet activation	27	0.0299
	response to wounding	18	0.0324
	negative thymic T cell selection	7	0.0348
	negative regulation of T cell activation	7	0.0348
	regulation of cytokine secretion	7	0.0348
	positive regulation of MAPK cascade	21	0.0373
	cellular calcium ion homeostasis	23	0.0384
	lymphocyte chemotaxis	11	0.0391
	cellular response to organic cyclic compound	17	0.0408
	tumor necrosis factor-mediated signaling pathway	27	0.0408
	ureteric bud development	13	0.0408
	negative regulation of tumor necrosis factor production	13	0.0408

	cGMP-mediated signaling	6	0.0420
	single organismal cell-cell adhesion	24	0.0482
CC	plasma membrane	721	0.0000
	extracellular region	358	0.0000
	external side of plasma membrane	100	0.0000
	integral component of plasma membrane	306	0.0000
	extracellular space	290	0.0000
	blood microparticle	54	0.0000
	cell surface	123	0.0000
	integral component of membrane	723	0.0000
	proteinaceous extracellular matrix	70	0.0000
	membrane raft	57	0.0000
	extracellular exosome	416	0.0000
	immunoglobulin complex, circulating	13	0.0000
	extrinsic component of cytoplasmic side of plasma membrane	25	0.0000
	T cell receptor complex	12	0.0000
	endocytic vesicle membrane	23	0.0001
	MHC class II protein complex	12	0.0003
	immunological synapse	15	0.0003
	receptor complex	33	0.0003
	mast cell granule	11	0.0012
	apical plasma membrane	57	0.0015
	anchored component of membrane	28	0.0036
	transport vesicle membrane	14	0.0048
	collagen trimer	24	0.0049
	sarcolemma	22	0.0105
	ruffle membrane	21	0.0164
	extrinsic component of external side of plasma membrane	6	0.0182
	integral component of lumenal side of endoplasmic reticulum membrane	11	0.0203
	neuron projection	44	0.0266
	caveola	17	0.0424
MF	antigen binding	64	0.0000
	serine-type endopeptidase activity	92	0.0000
	receptor activity	63	0.0000
	immunoglobulin receptor binding	19	0.0000
	receptor binding	81	0.0000
	heparin binding	45	0.0000
	Ras guanyl-nucleotide exchange factor activity	35	0.0000
	phosphatidylinositol-4,5-bisphosphate 3-kinase activity	24	0.0000
	transmembrane signaling receptor activity	51	0.0001
	MHC class II receptor activity	11	0.0001

	chemokine activity	20	0.0001
	calcium ion binding	124	0.0001
	extracellular matrix structural constituent	22	0.0009
	cytokine activity	40	0.0025
	cell adhesion molecule binding	20	0.0030
	transcriptional activator activity, RNA polymerase II core promoter proximal region sequence-specific binding	49	0.0030
	calcium-dependent phospholipid binding	19	0.0036
	lipopeptide binding	7	0.0037
	ATPase activity, coupled to transmembrane movement of substances	16	0.0041
	protein tyrosine kinase activity	32	0.0043
	Wnt-protein binding	13	0.0050
	non-membrane spanning protein tyrosine kinase activity	16	0.0064
	transmembrane receptor protein tyrosine kinase activity	14	0.0102
	integrin binding	26	0.0125
	SH3/SH2 adaptor activity	17	0.0187
	carbohydrate binding	39	0.0312
	virus receptor activity	19	0.0312
	drug binding	20	0.0313
	glucose transmembrane transporter activity	8	0.0323
	tumor necrosis factor-activated receptor activity	10	0.0372
	transcription regulatory region DNA binding	41	0.0372

Supplementary table 2 Significantly enriched KEGG

Term	Count	FDR
hsa04060:Cytokine-cytokine receptor interaction	82	0.0000
hsa04514:Cell adhesion molecules (CAMs)	58	0.0000
hsa04640:Hematopoietic cell lineage	39	0.0000
hsa05332:Graft-versus-host disease	22	0.0000
hsa04672:Intestinal immune network for IgA production	25	0.0000
hsa05330:Allograft rejection	22	0.0000
hsa04940:Type I diabetes mellitus	23	0.0000
hsa05144:Malaria	24	0.0000
hsa05323:Rheumatoid arthritis	33	0.0000
hsa05166:HTLV-I infection	65	0.0000
hsa05340:Primary immunodeficiency	19	0.0000
hsa04062:Chemokine signaling pathway	52	0.0000
hsa05416:Viral myocarditis	25	0.0000
hsa04668:TNF signaling pathway	36	0.0000
hsa05150:Staphylococcus aureus infection	24	0.0000
hsa05321:Inflammatory bowel disease (IBD)	26	0.0000

hsa04380:Osteoclast differentiation	39	0.0000
hsa05320:Autoimmune thyroid disease	22	0.0000
hsa04151:PI3K-Akt signaling pathway	73	0.0000
hsa04064:NF-kappa B signaling pathway	28	0.0000
hsa05310:Asthma	15	0.0000
hsa04024:cAMP signaling pathway	48	0.0000
hsa04660:T cell receptor signaling pathway	30	0.0000
hsa04510:Focal adhesion	49	0.0000
hsa05146:Amoebiasis	31	0.0000
hsa05200:Pathways in cancer	79	0.0000
hsa05142:Chagas disease (American trypanosomiasis)	30	0.0001
hsa05143:African trypanosomiasis	15	0.0001
hsa04512:ECM-receptor interaction	26	0.0002
hsa04020:Calcium signaling pathway	42	0.0002
hsa04145:Phagosome	37	0.0002
hsa05140:Leishmaniasis	22	0.0004
hsa05145:Toxoplasmosis	29	0.0005
hsa04630:Jak-STAT signaling pathway	35	0.0006
hsa05169:Epstein-Barr virus infection	31	0.0006
hsa04014:Ras signaling pathway	48	0.0007
hsa04662:B cell receptor signaling pathway	21	0.0007
hsa03320:PPAR signaling pathway	20	0.0014
hsa05414:Dilated cardiomyopathy	23	0.0016
hsa04610:Complement and coagulation cascades	20	0.0020
hsa02010:ABC transporters	15	0.0022
hsa05205:Proteoglycans in cancer	42	0.0022
hsa05162:Measles	31	0.0023
hsa04612:Antigen processing and presentation	21	0.0024
hsa04650:Natural killer cell mediated cytotoxicity	29	0.0025
hsa05152:Tuberculosis	38	0.0025
hsa04974:Protein digestion and absorption	23	0.0026
hsa04725:Cholinergic synapse	27	0.0026
hsa04923:Regulation of lipolysis in adipocytes	17	0.0029
hsa05410:Hypertrophic cardiomyopathy (HCM)	21	0.0030
hsa04713:Circadian entrainment	24	0.0030
hsa04920:Adipocytokine signaling pathway	19	0.0050
hsa05020:Prion diseases	12	0.0057
hsa04921:Oxytocin signaling pathway	32	0.0069
hsa05412:Arrhythmogenic right ventricular cardiomyopathy (ARVC)	18	0.0074
hsa04621:NOD-like receptor signaling pathway	16	0.0074
hsa04664:Fc epsilon RI signaling pathway	18	0.0085
hsa05164:Influenza A	35	0.0106

hsa04611:Platelet activation	28	0.0111
hsa04911:Insulin secretion	20	0.0174
hsa05222:Small cell lung cancer	20	0.0174
hsa05202:Transcriptional misregulation in cancer	33	0.0174
hsa04970:Salivary secretion	20	0.0195
hsa04015:Rap1 signaling pathway	39	0.0217
hsa05133:Pertussis	18	0.0217
hsa04022:cGMP-PKG signaling pathway	31	0.0237
hsa04924:Renin secretion	16	0.0237
hsa05214:Glioma	16	0.0271
hsa04666:Fc gamma R-mediated phagocytosis	19	0.0299
hsa00590:Arachidonic acid metabolism	15	0.0347
hsa04310:Wnt signaling pathway	27	0.0380
hsa04261:Adrenergic signaling in cardiomyocytes	27	0.0380
hsa05223:Non-small cell lung cancer	14	0.0380
hsa04960:Aldosterone-regulated sodium reabsorption	11	0.0392
hsa04012:ErbB signaling pathway	19	0.0392
hsa04925:Aldosterone synthesis and secretion	18	0.0399
hsa04080:Neuroactive ligand-receptor interaction	47	0.0410
hsa04723:Retrograde endocannabinoid signaling	21	0.0430
hsa04724:Glutamatergic synapse	23	0.0430
hsa04670:Leukocyte transendothelial migration	23	0.0466
hsa04066:HIF-1 signaling pathway	20	0.0483
hsa05168:Herpes simplex infection	33	0.0485
